# A pathogen's spatial range is not constrained by geographical features in the flax rust pathosystem

**DOI:** 10.1002/ece3.10577

**Published:** 2023-10-09

**Authors:** Keenan Duggal, Ian Miller, Juliana Jiranek, Jessica Metcalf

**Affiliations:** ^1^ Department of Ecology and Evolutionary Biology Princeton University Princeton New Jersey USA; ^2^ Rocky Mountain Biological Laboratory Gothic Colorado USA; ^3^ Department of Biology University of Virginia Charlottesville United States

**Keywords:** climate change, flax rust, Lewis flax, plant pathogens, range shifts, spatial range

## Abstract

Climate change and shifting environmental conditions can allow pathogens to spread into previously unburdened areas. For plant pathogens, this dynamic has the potential to disrupt natural ecosystem equilibria and human agriculture, making predicting plant pathogen range shifts increasingly important. Although such predictions will hinge on an accurate understanding of the determinants of pathogen range—namely the environmental, geographical, and host range characteristics that modulate local pathogen habitation—few studies to date have probed these in natural plant populations. Here, we characterize range determinants for the model system of Lewis flax (*Linum lewisii*) and its pathogen, flax rust (*Melampsora lini*), in the Rocky Mountains. Transect surveys were performed to assess three relationships: (i) the effect of geographical features—elevation, slope aspect, slope grade, and land cover—on flax presence and density, (ii) the effect of geographical features on flax rust presence and prevalence, and (iii) the effects of flax's local population density and metapopulation structure on flax rust presence and prevalence. We found that flax population density, but not host metapopulation structure, influences the distribution of flax rust. Additionally, we showed that, while the distribution of flax was broadly constrained to a relatively narrow range of geographical and resulting environmental features, flax rust was evenly distributed across the full range of settings measured. These results indicate that a warming environment, which is expected to modulate such features, may restrict the optimal range of the plant more than that of its pathogen. Importantly, our results also suggest that even if flax shifts its spatial range to escape increasing climatic pressures, flax rust will not face any significant barriers to track this movement.

## Graphical abstract

Climate change will induce shifts in the spatial ranges of plants and their pathogens. This article addresses some of the key physical features of the environment that might modulate such movement dynamics, thereby influencing epidemiological outcomes.

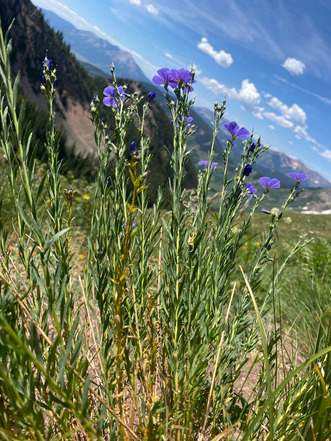

## 1 INTRODUCTION

Plant pathogens play a critical role in shaping natural ecosystems. Although often found in low abundance (Dinoor & Eshed, 1984; Höckerstedt et al., 2022), plant pathogens can modulate the fitness of host populations and promote ecosystem species diversity (Gilbert, 2002; Mordecai, 2011; Termorshuizen, 2016). However, when pathogens escape their endemic ranges and encounter naïve hosts, they can destabilize ecosystems (Crowl et al., 2008; Dinoor & Eshed, 1984). Accordingly, climate change could pose a serious threat to the health of ecosystems by exacerbating plant pathogen movement and epidemics (Chen et al., 2011). The potential for increased spillover events from existing natural plant pathogen reservoirs is particularly problematic for mono‐cultured crop agroecosystems, which already face yield losses of around 20%–30% due to plant pathogens (McCann, 2020; Power & Mitchell, 2004; Savary et al., 2019).

Characterizing the degree to which plant pathogens are directly constrained by environmental, geographical, and ecological factors is necessary to ground expectations for the scope of future impacts. By virtue of their obligate parasitism, many plant pathogens cannot exist without their host, and their range thus must entirely overlap with that of their host. However, a plant pathogen's range can be tapered into a smaller subset of its host plant's range (Shaw & Osborne, 2011). When this is the case, there exists a potential for changing environmental conditions to drive novel disease establishment into previously unburdened areas (Bebber et al., 2013). Similarly, if plant pathogen performance is locally inhibited anywhere in its host's range, then changing conditions could drive up infection intensity and prevalence in the previously suppressed regions. Additionally, as climate change continues to induce range shifts for plants in natural plant pathosystems (Kelly & Goulden, 2008), each systems' pathogens could respond with either equivalent shifts or incomplete shifts, and the systems' epidemiological dynamics could be affected. Determining which conditions constrain plant pathogen presence and performance will open the way to predicting how climate change might affect future plant pathogen dynamics and regional disease burdens.

Specific climate and weather conditions have been shown to affect plant pathogen performance (Shaw & Osborne, 2011; Van Den Berg & Van Den Bosch, 2007), with evidence that plant pathogens have relatively narrow optimal tolerances for rain, humidity, temperature, and carbon dioxide levels (Trecate et al., 2019; Velásquez et al., 2018). An important determinant of the local climatic conditions experienced by plants and their pathogens is their physical geographical environment. For instance, topography and slope characteristics are both known to dictate regional variations in temperature and precipitation (Nyman et al., 2018). Despite the importance of geography to microclimate patterning, very few studies have characterized whether large‐scale geographical features of the environment constrain the range and performance of plant pathogens, and there is a scarcity of field data that can be used to inform pathogen movement modeling efforts (Abbate & Antonovics, 2014; Bruns et al., 2019; Shaw & Osborne, 2011).

Additionally, the landscape distribution of plants can affect plant pathogen dynamics (Opdam & Wascher, 2004; Shaw & Osborne, 2011). Previous studies have shown that host spatial distribution strongly correlates with infection levels, with population size and metapopulation connectivity both modulating infection intensity (Jousimo et al., 2014; Laine & Hanski, 2006; Papaïx et al., 2021). Moreover, it has been shown that plant population densities can correlate with both higher and lower incidences of infection (Burdon & Chilvers, 1982). Accordingly, it is possible that host population and metapopulation characteristics may set limits on pathogen range extent and local performance levels.

To characterize how host population, host metapopulation, and geographical landscape features modulate the spatial range and performance of plant pathogens, we studied Lewis flax (*Linum lewisii*) and its fungal pathogen, flax rust (*Melamspora lini*) in the Rocky Mountains. Epidemiological results from this system are particularly relevant because *L. lewisii* is a close genetic relative of agricultural flax (Ahmed et al., 2018), for which *M. lini* is known to be a significant pest (Lawrence et al., 2007). Additionally gaining insight into this fungal plant pathogen system is valuable on a larger scale because phytopathogenic fungi have significant impacts on ecosystem epidemics and on agricultural crop yields (Knogge, 1996).

Over the course of the 2021 summer field season, we characterized the distributions of flax and flax rust across transects that captured the extent of four ecogeographical features—elevation, slope aspect, slope grade, and land cover. The steep temperature gradient at high altitudes allows us to indirectly study broad thermal constraints on flax and flax rust range and density, with the prediction of consistent patterns across the two species given their history of coevolution. Slope aspect and grade also shape plant community composition via effects on daily sun exposure and local soil characteristics and on wind exposure and dispersal dynamics, respectively (Rahman et al., 2022; Singh, 2018). As local ecosystem composition will also affect plant, and potentially pathogen performance, we modeled the effects of land cover as a broad approximation of local ecosystem on flax and flax rust presence and density. Finally, we recorded the density and coordinates of each population to probe for effects of population density and connectivity on local pathogen burden. We predicted that populations with high local densities of flax situated close to neighboring flax populations would display higher disease burdens as a result of local and global transmission, respectively.

## 2 MATERIALS AND METHODS

### 2.1 Study system

Lewis flax is a perennial sub‐alpine wildflower found throughout the mountainous regions of western North America (Innes et al., 2022). Flax rust is a wind‐dispersed fungal pathogen (Ravensdale et al., 2011) that carries out all life stages on living flax tissue (Lawrence et al., 2007). Although the distribution of *M. lini* in North America has not been described extensively, it is known to infect other species in the Linaceae family across their ranges (e.g., Innes et al., 2022; Springer, 2009). However, *L. lewisii* is the only species in Linaceae found appreciably in the Gunnison Valley (USDA, NRCS, 2023), and is thus necessarily responsible for the epidemiological dynamics in this region.

Mechanistically, rust extracts nutrients from flax upon infection, which can cause reductions in flax seed yield and deleterious defoliation (Lawrence et al., 2007). Rust infections can be perennial, with the fungi overwintering in the root system of the plant and reemerging to begin its sporulating phase once conditions are favorable in the spring (Lawrence et al., 2007; Susi et al., 2020). However, in our study system, rust appears to overwinter successfully on infected plants in no more than ~20% of cases (Duggal, K., & Jiranek, J. unpublished data).

### 2.2 Transect selection

**FIGURE 1:**
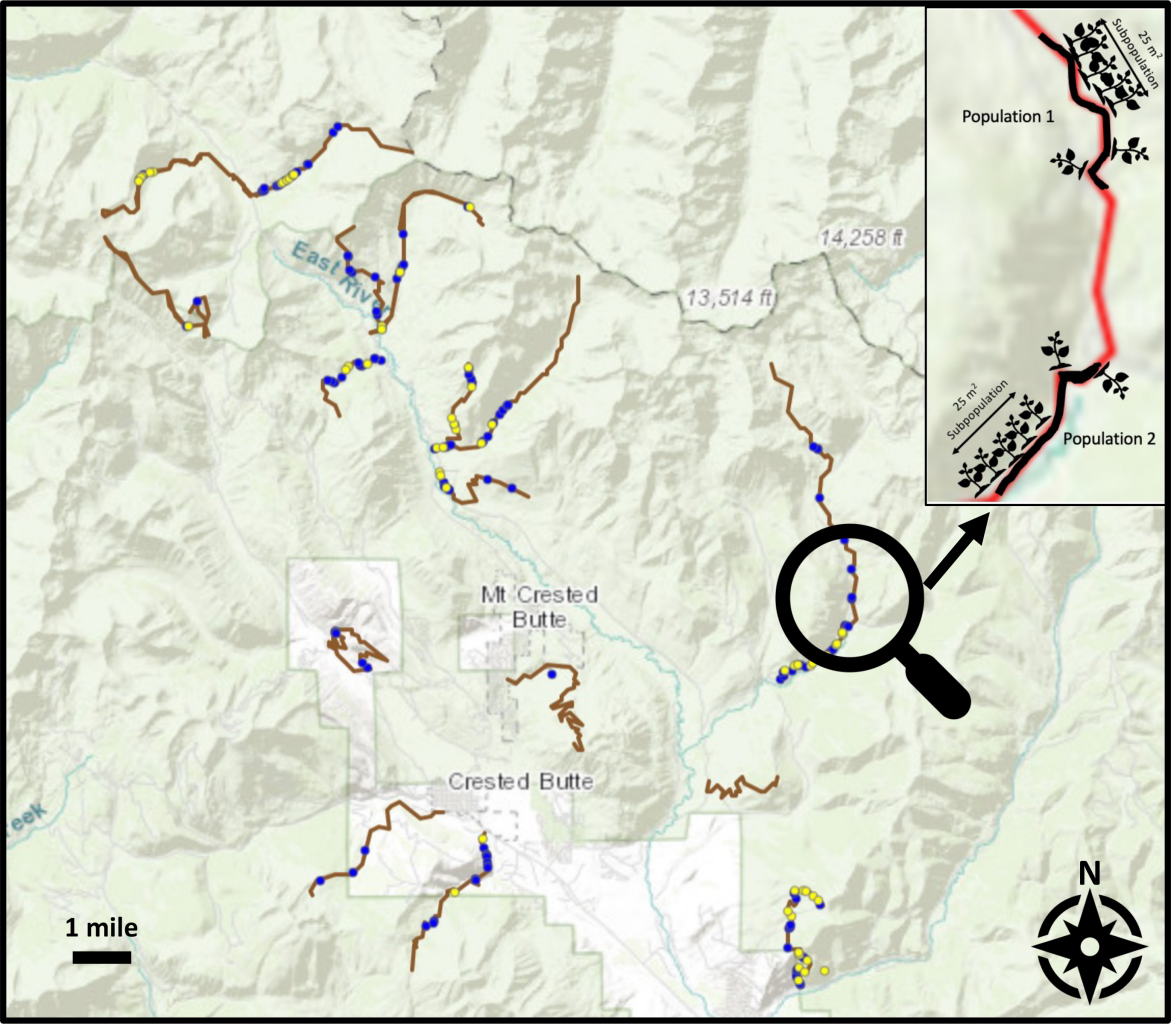
Map of the surveyed flax and rust locations in the Upper Gunnison Valley. Seventeen transects that were traversed in the greater Upper Gunnison River Basin area during the 2021 field season were used for analyses. Transect routes are represented with brown lines. Healthy populations are depicted in blue; diseased are shown in yellow.

The study was performed in the area surrounding the Rocky Mountain Biological Laboratory in the Gunnison Valley in Colorado (Figure 1). Transects were systematically selected from the Upper Gunnison Basin's established hiking trails to cover the diversity of land cover and topography of the region. Trails that spanned large elevation gains and losses and trails that captured significant variation in slope aspect and grade were prioritized.

### 2.3 Transect sampling

Twenty‐three total elevation transects were traversed by foot. For each transect, the coordinates of the transect line and the precise latitudes and longitudes bounding every flax population were recorded. An observational area consisting of 2.5 m to either side of the trail was continuously scrutinized for the presence of flax as the transect was being performed. Whenever flax was found in a density ≥5 plants in a 25‐m^2^ area, the existence of a population was noted and the coordinates of the first flax plant were recorded. The end of the population and its coordinates were recorded after no flax was observed for 10 m on either side of the trail.

Because our focus was not on quantifying the precise disease characteristics within single populations (e.g., as in Abbate & Antonovics, 2014), but rather on capturing larger geographical features that modulate disease presence and prevalence, we sampled plant population density and disease prevalence from fixed 25 m^2^ subpopulations at the beginning of each population. This methodology was designed to maximize the number of transects we could visit, enabling us to cover a larger span of geographical settings. Additionally, all flax plants in each population outside of the 25 m^2^ subpopulation region were examined for the presence of flax rust. If any flax rust was observed in the larger population, the “presence of flax rust” was noted as 1, and 0 otherwise. In summary, three aspects of each local flax—rust pathosystem were quantified: the total number of flax plants in a 25‐m^2^ subpopulation, the number of diseased flax plants in the 25‐m^2^ subpopulation, and the binary presence/absence of rust in the entire population. In all subsequent analyses: rust *prevalence* refers to the ratio of the number of subpopulation diseased plants/the total number of plants in each 25 m^2^ subpopulation, and rust *presence* refers directly to the binary rust presence/absence recorded for each whole population.

The sampling for this study was performed over the course of a single growing season to prevent extra‐seasonal epidemiological and ecological dynamics from confounding the results. A consequence of this methodology is that it necessitated surveying transects at different dates throughout the summer, thereby introducing temporality as a possible confounder. While the presumed rarity of cross‐population disease transmission makes it unlikely that disease presence measurements were biased by within‐season temporality, measures of prevalence might have been affected. Two precautions were taken to mitigate an effect of temporality on prevalence measurements. First, the inspection of each subpopulation for disease concentrated exclusively on highly diseased flax plants rather than on those containing a small number of obscure, emerging disease pustules. While this approach likely caused the prevalence measurements to underestimate each population's true prevalence, it ensures that recently diseased plants were excluded, reducing bias resulting from recent spread in populations sampled later in the season. Second, each transect was visited in an order such that values of each regressed ecogeographical variable were distributed approximately evenly across time (Appendix 5). Despite the evidence suggesting that these precautions were relatively effective (Appendix 1), the prevalence data in this article should be treated as semiquantitative approximations, rather than exact measurements of population level disease burden.

### 2.4 Data processing

The GPS file for each transect was cleaned using a GPX Editor to eliminate irregular path deviations (e.g., from backtracking) that were inconsistent with the linear progression of the trail. A rectangular ribbon extending 2.5 m to either side of the trail was created, and points were regularly sampled from the ribbon (1/m^2^). Then, using the flax population GPS coordinates (with the population end coordinates set back by 10 m to account for the sampling methodology), each sampled point was classified as either no flax, healthy flax, or diseased flax. Additionally, the distance from a population to the nearest neighboring population and to the nearest diseased population was calculated.

The Rocky Mountain Biological Laboratory spatial datasets encoding raster data for elevation, slope aspect, slope grade, and land cover were aggregated in R (Breckheimer, 2022). For additional analyses, we used the PRISM Climate Group's 30‐year average temperature raster data with 800 m spatial resolutions (Daly & National Center for Atmospheric Research Staff, 2020). For each sampled point location, the raster data from each of the spatial datasets was extracted and included for analyses. For each population, the mean elevation, slope aspect and slope grade, mode land‐cover type, and average annual temperature were extracted. Ultimately, only 17 (172 populations) of the original 23 transects (244 flax populations) fell entirely into the geographical bounds of the spatial datasets, and only these were used for analyses in this article.

### 2.5 Data analysis

To accommodate expected nonlinear relationships within the data, we fit generalized additive models, with a logit link function for binary variables (e.g., flax or flax rust presence/absence), using the “mgcv” package in R (4.2.2) (Wood, 2011). For each statistical analysis, we used the gam.check() function, and independently evaluated the distribution of deviance residuals to establish that conditions for regression were met.

First, we fit a generalized additive model to characterize determinants of flax presence. We regressed flax presence against elevation, slope grade, land cover, and slope aspect (the RMBL Spatial Data Platform has two slope aspect raster datasets encoding slope aspect: one for the degree of slope “westernness” and one for slope “southerness”). Second, we fit two GAM models to characterize determinants of flax rust presence and prevalence. We tested the same predictors as those used for flax presence while also including flax population density. Third, we used the R package “spdep” to create nonparametric spatial autocorrelation correlograms to visualize the spatial correlation of flax rust presence and prevalence up to 30 m away from a population's starting coordinates (Bivand & Wong, 2018). As an additional measure to determine spatial correlation of rust frequency, we performed 1000 Monte Carlo simulations of Moran's test on both flax rust presence and prevalence using the R package “spdep.” For this test, we set a larger radius of what constituted the farthest functional “neighbors” of a given flax population at 250 m.

Finally, we leveraged the PRISM Climate Group's 30‐year average annual temperature public raster dataset. The resolution of the PRISM temperature dataset had a significantly lower spatial acuity (800 m) compared to the RMBL spatial datasets (1–3 m). Accordingly, rather than using the temperature data as a direct estimate of a plant or population's local microclimate, we instead used it to characterize a general relationship between temperature and elevation at our flax population locations. Model fitting criteria identified a fourth degree polynomial model as the best model to describe the relationship between temperature and elevation across each of the flax population locations.

## 3 RESULTS

### 3.1 Flax is constrained by geographical features of the environment

**FIGURE 2:**
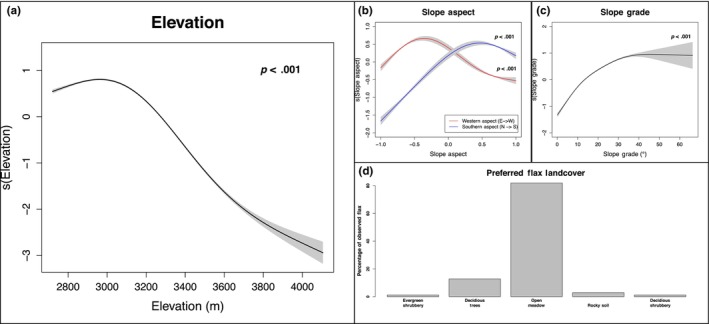
Ecogeographical predictors of flax presence. (a–d) A GAM model of flax presence is shown with five predictor terms: elevation, western slope aspect, southern slope aspect, slope grade, and land cover. Shaded regions represent ±2 standard errors of the mean. (a) Fitted smooth of the elevation predictor variable. Because we only studied Lewis flax at relatively high elevations in the Upper Gunnison River Basin, this range should not be interpreted as fixed biological limits, but rather as an indication that flax is locally most competitive under a particular set of altitude‐associated conditions. (b) Fitted smooth of the slope aspect predictor. Western slope aspect (east = −1 → west = 1) is depicted in red and the southern slope aspect (north = −1 → south = 1) is depicted in blue. (c) Fitted smooth of the slope grade predictor. (d) The land‐cover classes containing flax are depicted with their proportion of the total observed flax. Singular instances of flax in lower frequency land‐cover classes are not depicted because land‐cover data were encoded as the modal value of each population.

The fitted model of flax presence indicates that the optimal elevation range for flax lies approximately between 2900 and 3100 m, with a drop off in likelihood of presence at lower elevations and an even greater decline at higher elevations (*p* < 2 × 10^−16^; Figure 2a). Because we found that elevation has little bearing on flax population density (Appendix 2), we postulate that this altitudinal constraint of flax is defined by limits to establishment rather than a continuum of preferable conditions for flax. While flax distributions do not seem to be strongly influenced on either end of the East–West slope aspect axis, there is a fairly strong correlation between flax presence and southern facing slopes (*p* < 2 × 10^−16^; Figure 2b). Additionally, the model reveals that flax is found preferentially in areas with steeper slopes up to a certain point (~40°; *p* < 2 × 10^−16^; Figure 2c). Finally, we investigated whether flax is found preferentially in certain classes of land cover over others. Of the 11 different types of land cover that we surveyed (Table 1), flax is found primarily in open meadows and secondarily in deciduous forests, rocky soil terrains, and evergreen shrubbery (Figure 2d).

**TABLE 1:** Proportion of total flax and rust‐infected flax in each land‐cover category^a^.

	Open meadow	Deciduous trees	Rocky soil	Evergreen shrubbery	Deciduous shrubbery
Total flax	0.82	0.13	0.03	0.01	0.01
Proportion of infected flax					
All infected	0.85	0.11	0.02	0.02	0
1st Tercile prevalence	1.0	0	0	0	0
2nd Tercile prevalence	0.70	0.20	0.10	0	0
3rd Tercile prevalence	0.70	0.20	0	0.1	0

^a^ Categories that held negligible flax: persistent open water, building or structure; paved or other impervious surface; irrigated pasture; evergreen forest understory; deciduous forest understory.

### 3.2 Flax rust is not constrained by the same ecogeographical conditions

**FIGURE 3:**
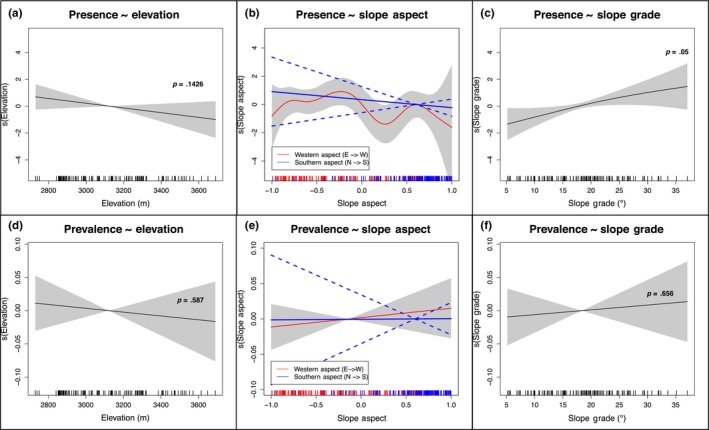
Geographical effects on flax rust range and performance. (a–c) A GAM model for flax rust presence is shown with six predictor terms: elevation, western slope aspect, southern slope aspect, slope grade, land cover, and flax density. (d–f) A GAM model for flax rust prevalence is shown with the same six predictors. Shaded regions represent ±2 standard errors. (a, d) Fitted smooth for the elevation predictor variable. (b, e) Fitted smooth for the western slope aspect (E→W; red) and southern slope aspect (N→S; blue) predictors. (c, f) Fitted smooth for the slope grade predictor variable.

Of the 172 analyzed flax populations, 55 contained flax rust within the larger population and 30 contained flax rust within the populations' 25 m^2^ surveyed subpopulation area. The fitted model of flax rust presence showed no significant effect of elevation (*p* = .143; Figure 3a). Similarly, no significant relationship was seen between elevation and flax rust prevalence (*p* = .587; Figure 3d). No significant relationships were observed between E‐W or N‐S slope aspect and flax rust presence (*p* = .328, *p* = .455; Figure 3b) or prevalence (*p* = .482, *p* = .976; Figure 3e). While there was a significant positive effect of slope grade on disease presence (*p* = .046; Figure 3c), there was no corresponding relationship between slope grade and disease prevalence (*p* = .656; Figure 3f). Finally, we analyzed the modal land‐cover classes that contained flax rust and unsurprisingly found that rust presence is most commonly observed in the same land‐cover classes that contain the most flax (Table 1). We further analyzed the proportion of diseased flax plants contained in each land‐cover category for the three terciles of disease prevalence within infected populations. While there were minor differences in the different prevalence classes (Table 1), these can likely be attributed to small sample sizes in each prevalence class (*n* = 10).

### 3.3 Flax rust infection is determined by population density, not metapopulation connectivity

**FIGURE 4:**
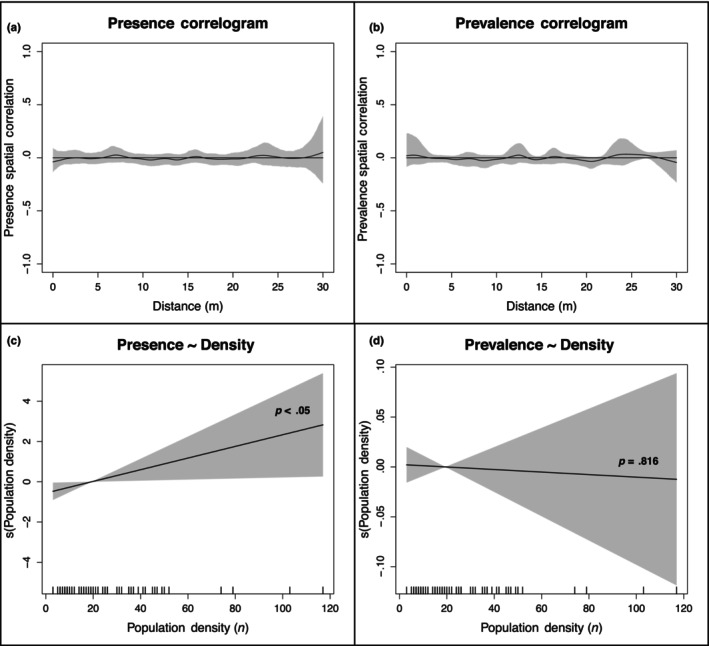
Effects of flax metapopulation connectivity and population density on flax rust disease characteristics. Non‐parametric correlograms show the spatial correlation of flax rust (a) presence and (b) prevalence. Fitted smooth for the population density predictor in the GAM model of flax rust (c) presence and (d) prevalence.

To determine whether flax metapopulation structure influences the distribution of flax rust, we first parameterized metapopulation structure as the distance between a population and its neighboring populations to capture connectivity (Taylor et al., 1993). Nonparametric spatial correlograms show no significant spatial correlation of flax rust presence (Figure 4a) or prevalence (Figure 4b) for immediate population neighbors (here we ignored low‐frequency long‐distance dispersal events of >30 m). Given the lack of spatial correlation for disease among immediate neighbors, we did not expect to see any spatial correlation among more separated populations. To confirm this, we performed 1000 Monte Carlo simulations of Moran's test for flax rust presence and prevalence with functional “neighbors” parameterized as populations within 250 m of a central flax population. We found no significant spatial correlation for flax rust presence (statistic = −.025111, *p* = .601) or prevalence (statistic = −.039276, *p* = .711).

Interestingly, although we did not find flax rust disease presence to be correlated with flax population connectivity, it does appear to be correlated with local flax population density (Figure 4c). Specifically, there is a strong positive correlation between host plant density and flax rust presence (*p* = .028), but not with rust prevalence (*p* = .816; Figure 4d). Additionally, a Student's *t* test indicates that high‐density populations (density > mean density) show a significant increased presence of flax rust relative to low‐density populations (density < mean density; *p* = .0124). All else equal, increasing numbers of plants provide more opportunities for pathogen establishment, such that a linear relationship is expected. A slope greater than one would indicate higher transmission in high‐density populations. We tested for this by simulating expectations under the null. Ultimately, a Student's *t* test indicates that the slope is not significantly greater than one (*p* = .676), meaning that denser populations present more infection opportunities because of their higher availability of susceptible plant tissue, not because of any density‐specific pathogen facilitation.

### 3.4 Forecasting climate change impacts on flax

The optimal ranges of plant species in mountain ecosystems are expected to shift upward in elevation due to climate change (Gottfried et al., 2012; Lenoir et al., 2008). With precedents of both successful and unsuccessful range shifts due to climate change in the area of our study, some plants will have more success than others at shifting upward (Brown & Vellend, 2014; Miller‐Rushing & Inouye, 2009; Pyke et al., 2016). Combined with our transect data, the RMBL spatial datasets offer a unique opportunity to explore whether flax will be capable of migrating with climate change.

**FIGURE 5:**
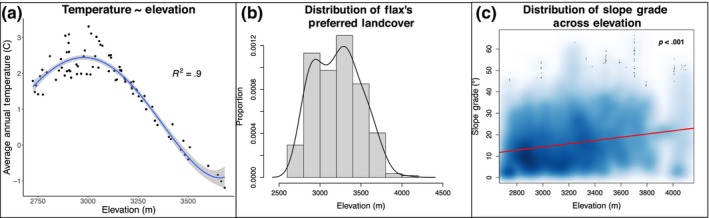
Forecasting effects of climate change on flax. (a) A fourth degree polynomial regression of elevation on annual mean temperature was created using observed flax population location data. (b) The distribution of open grassy meadow, flax's preferred land cover, across our transects is plotted in a histogram with a density line overlaid. (c) Slope grade is plotted across elevation for all observed transect points with an overlaid linear regression fit (red).

In order to establish prospects for flax spread, we first mapped temperature to elevation in our study area to confirm that global warming is likely to result in flax‐suitable temperatures at higher altitudes (Figure 5a) and then we evaluated whether other requirements for flax establishment are present at these altitudes. We fit a fourth degree polynomial curve (Figure 5a) describing the relationship between temperature and elevation for flax population locations:

$$\begin{array}{c} T=1.23\times 10^{3}-1.72\left(E\right)+8.88\times 10^{-4}\left(E^{2}\right)\\ \;-2.01\times 10^{-7}\left(E^{3}\right)+1.67\times 10^{-11}\left(E^{4}\right) \end{array}$$

Pr>|t|=.023,.013,.007,.004,.002

AdjustedR−squared=.87.

While we only analyzed the relationship between elevation and mean annual temperature, thus obscuring any effects that shorter time‐scale temperature patterns might have on living organisms, these data show that flax will be able to escape climate change‐induced stress by shifting upward. Additionally, we found that open grassy meadow, the preferred land cover of flax (Figure 5b) and the optimal grades in slope (~30°; Figure 5c) are both present at the higher elevations into which flax would migrate. Unsurprisingly, we found that slope aspect was more or less evenly distributed across elevation (Appendix 3).

## 4 DISCUSSION

The ecogeographical limits of the ranges of hosts and pathogens, their overlap, and the detail of how host population structure modulates pathogen spread will all determine pathogen impact in the context of a changing climate. We applied statistical models and a null model of epidemiological spread to data on the flax rust system from a landscape in the Rocky Mountains to investigate abiotic and biotic factors driving the antagonistic interaction. Ecogeographical constraints identified for the host were not apparent for the pathogen. Boundaries on pathogen distribution are thus largely set by the ecogeographical limits on the host. Lower host density reduces pathogen presence, but not to a degree beyond that expected by the reduction in the number of susceptible plants; and the spatial structure of the host plant population also does not seem to constrain pathogen spread. All of this suggests that the pathogen range could easily expand to encompass any extension of the host plant.

### 4.1 Flax may experience a net range contraction as a result of climate change

The distribution of flax was broadly constrained across elevations between 2900 and 3100 m. A narrow optimal elevation range accords with results showing that plant species are on average less adapted to the biotic and abiotic conditions at the peripheries of their ranges (Angert et al., 2020; le Roux et al., 2012). The lack of correlation between flax population density and elevation might reflect local adaptation in peripheral populations (Appendix 2, Sork, 2018) or equalizing competition yielding similar densities across a range of environmental settings. Flax concentration on southern facing slopes can be explained by the dependency of flax flowering on high‐intensity sunlight, which in the Northern hemisphere is concentrated on southern facing slopes (Addicott, 1977; Måren et al., 2015). That flax performs best on moderately steep slopes aligns with our observations that steeper slopes tend to contain shorter canopy vegetation, are more exposed to sunlight, and facilitate widespread seed dispersal. Finally, we show that flax performs best in select land‐cover categories, especially in grassy open meadows that receive significant sunlight. In addition to these tested ecogeographical features, the distribution of flax could be modulated by a variety of peripheral variables such as soil type (Springer et al., 2007) and mycorrhizal interactions (Tedersoo et al., 2020).

The narrow ecogeographical niche of flax indicates that climate change will pose several challenges for the plant species. Primarily, we predict that flax will become less competitive in the lower ranges of its current distribution. For plants, biotic competition is thought to govern the lower species distribution limits, and climate change is predicted to create novel biotic competition and pressures in these zones (Choler et al., 2001; Guisan & Theurillat, 2000; Kulonen et al., 2018; Pauli et al., 2007). Flax may adapt to these pressures with an upward range shift. While it is possible that flax could hit an abiotic wall preventing it from migrating upward (Brown & Vellend, 2014), several lines of evidence suggest that flax will be able to make this shift. In this study, we found that higher elevations contain suitable habitat for flax (Figure 5). Moreover, studies have shown that many plants are able to overcome novel biotic competition as they migrate upward (Alexander et al., 2015), and there have already been precedents of plant species shifting their ranges upward due to recent global warming (Telwala et al., 2013). However, climate change is unlikely to shift conditions at higher elevations to perfectly resemble those of lower elevations (Brown & Vellend, 2014), and there will likely be discontinuity in several abiotic factors like gas‐phase nutrients, solar radiation, aridity, and water availability which are thought to govern a species' upper range limit (Kulonen et al., 2018; Smith et al., 2008). Thus, even if flax can migrate upward, it will be confronted with new abiotic stressors, potentially resulting in further adaptation demands. This phenomenon has already been observed in a similar alpine plant system, in which upward migrating monkeyflower lineages experienced selection pressures for novel traits (DeMarche et al., 2020). Eventually, even if flax adapts to novel stressors, it will exhaust the supply of suitable higher elevation terrain into which it can migrate (Figure 5b; Elsen & Tingley, 2015). Taken together, all of these findings suggest that flax may experience a net range contraction (Auld et al., 2022).

### 4.2 The distribution of flax rust is unlikely to be directly modulated by climate change

On the other hand, our data show that, compared to flax, flax rust is relatively insensitive to ecogeographical features of its environment. In contrast to results from an anther‐smut wildflower system, we found that the presence and prevalence of flax rust are not affected by the same gradients of elevation and slope aspect that influence the range of flax (Abbate & Antonovics, 2014). Moreover, flax rust is found in almost the same proportions in each of the land‐cover categories as flax (Table 1), which suggests that ecosystem environments do not influence rust infection dynamics to any great extent. Because flax rust is observed evenly across the full range of ecogeographical features (Figure 3), it can be inferred that the viable geographical range of flax rust extends beyond its observable range, which is constrained by the current distribution of flax. This suggests that flax rust will be able to follow flax movement without facing inhibited performance due to climate change.

Our results are also surprising given that prior work has suggested that fungal pathogens have specific temperature and humidity optima (Clarkson et al., 2014; Lawrence et al., 2007; Miller et al., 2022). The decline in temperature and flax presence with elevation (Figures 2a and 5b) is not matched by an equivalent decline in pathogen presence or prevalence (Figure 3a,d). Thus, it appears as though the degree of climatic sensitivity for the pathogen is less acute than that of its plant host in natural settings. This finding is consistent with other fungal pathogen–plant systems (Cohen et al., 2017; Nowakowski et al., 2016; Rohr et al., 2018; Singh et al., 2023), and aligns with the previous finding that the abiotic fundamental niches of plant pathogens are often wider than their realized niches (Chaloner et al., 2020). It is important to note here that our survey methodology was designed to elucidate the broad influences of ecogeographical variables on flax and flax rust distributions, not to create precise models of how flax or flax rust will respond to climate change. It is possible that climate change will induce minor shifts in viable plant pathogen geographic ranges, but we show that any such changes are unlikely to be as dramatic as those for their plant hosts.

### 4.3 Metapopulation characteristics are not major drivers of local disease burdens

In terms of landscape‐level disease dynamics, our research shows that flax rust transmission is relatively independent of flax metapopulation connectivity and depends instead on population density. This finding is consistent with the previous studies (Burdon & Chilvers, 1982; Delmotte et al., 1999), and with intuition. Long‐distance stochastic wind dispersal events between populations separated by up to a kilometer, while an important factor in aerially dispersed pathogen life history, are far less frequent than short within‐population dispersal events (Rieux et al., 2014), and should therefore contribute less to local epidemic intensities. While our survey methodology does not perfectly capture population connectivity because we only surveyed an area of 5 m^2^ along quasi‐linear trails, it does provide a realistic approximation of how populations are spatially distributed. This finding suggests that even if climate change alters flax metapopulation structures, which our data suggests will not happen to any substantial degree (Appendix 4), this will probably not have significant effects on the transmission of flax rust within a local population over a growing season once disease establishes there.

### 4.4 Potential effects of climate change on flax rust epidemiological dynamics

**FIGURE 6:**
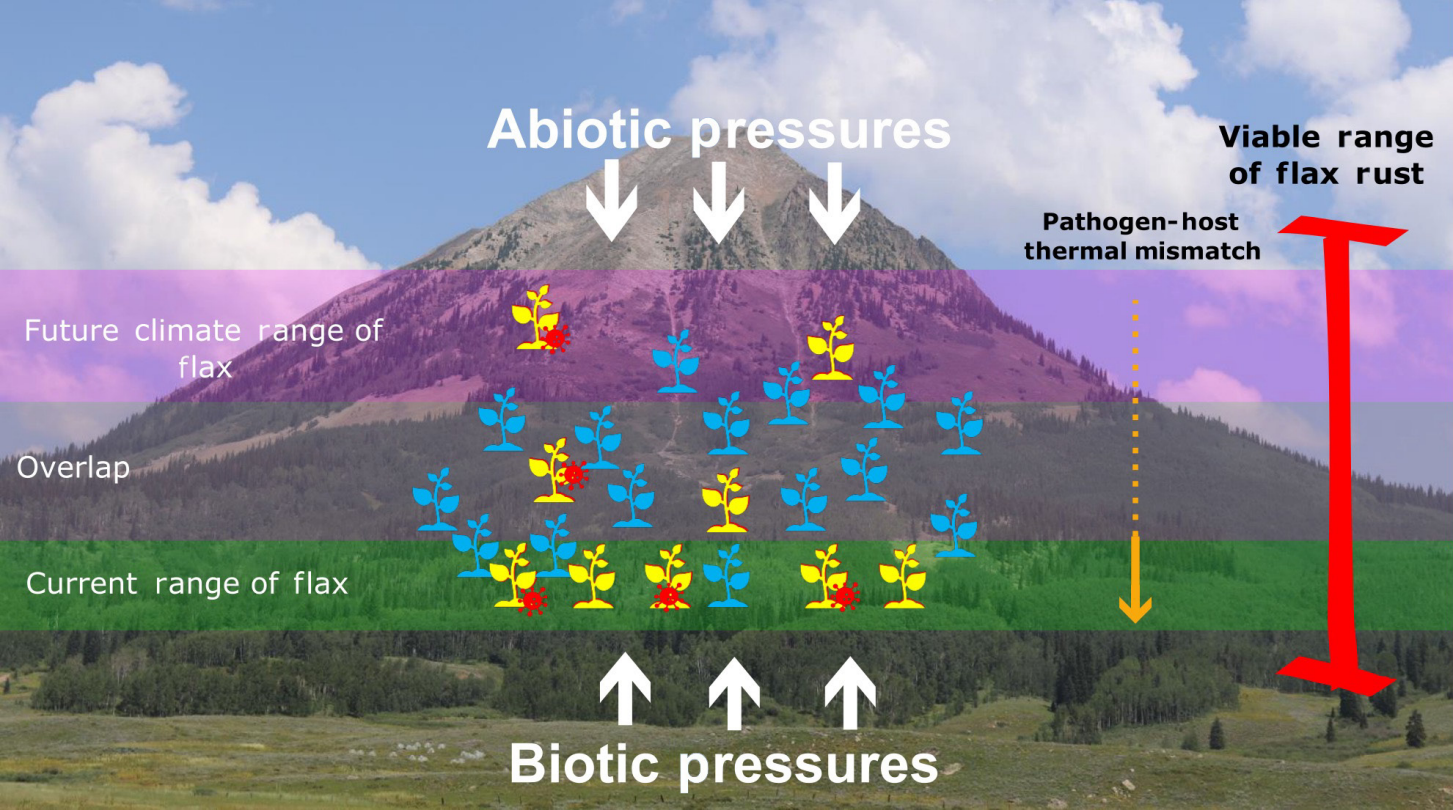
A schematic model of how climate change may affect the flax rust pathosystem. Yellow represents plants experiencing stress from either disease (red icon) or unsuitable climate conditions. Blue represents healthy plants (photo taken by Ryan Williams).

Collectively, our results suggest that flax will be adversely affected more than flax rust by the effects of climate change (Figure 6). In fact, climate change may actually *increase* rust performance in some flax populations. According to the Thermal Mismatch Hypothesis, a theory derived from fungal studies in amphibians, host susceptibility to pathogens increases as environmental conditions become less favorable (Cohen et al., 2017, 2020). It has not yet been established whether the TMH applies to plant–pathogen systems (Bruns et al., 2019; Dudney et al., 2021), but our data does support its key assumption: that pathogens have a greater implied thermal breadth than their hosts. A consequence of this theory is that climate change may drive up the prevalence and presence of rust infections in some flax populations. Even if there is space and flax is capable of migrating upward to escape suboptimal thermal microclimates (Figure 5), it is possible that there would be a temporal lag in which flax would still face elevated pathogen pressure (Alexander et al., 2018). And, even if there is no significant lag and flax is able to quickly migrate upward to escape novel biotic stresses, flax could still face higher vulnerability to flax rust. Prior studies in *Arabidopsis*, rice and trees have all shown inverse relationships between abiotic stress levels and plant disease resistance levels (Kolb et al., 2016; Xiong & Yang, 2003; Yasuda et al., 2008). On the other hand, some studies have shown that abiotic stress can actually prime plants' immune responses and make them *more* resistant to pathogens (Ramegowda & Senthil‐Kumar, 2015; Saijo & Loo, 2020). A more detailed mechanistic study will be needed to determine the actual dynamic in flax and related plants.

It is important to understand how climate change will affect plant pathogens. Here, we show that the distribution of flax is constrained by ecogeographical features of the environment, whereas its pathogen, flax rust, shows a broader tolerance for varying local environments. Additionally, we show that flax rust presence is driven primarily by within‐population variables like host population size, and less so by metapopulation structure. Finally, we show that, in terms of biotic and abiotic geographical features, there is suitable land at higher elevations to sustain a climate‐driven upward shift in the distribution of flax. From these results, we propose that wind‐dispersed fungal plant pathogens like flax rust will not face significant impediments to range shifts necessitated by host range shifts. This finding implies a possibility that flax rust, already adapted to new geographic and environmental conditions and widely dispersed through the wind, might affect flax establishment and subsequent epidemiological dynamics in novel flax habitats. Such patterns will need to be carefully monitored in flax and other plants with similar life histories moving forward.

## AUTHOR CONTRIBUTIONS

**Keenan Duggal:** Conceptualization (supporting); data curation (equal); formal analysis (lead); investigation (equal); methodology (equal); project administration (equal); visualization (lead); writing – original draft (lead); writing – review and editing (lead). **Ian Miller:** Conceptualization (lead); data curation (equal); formal analysis (supporting); funding acquisition (equal); investigation (equal); methodology (equal); project administration (equal); supervision (lead); writing – original draft (equal); writing – review and editing (equal). **Juliana Jiranek:** Conceptualization (supporting); formal analysis (supporting); writing – review and editing (supporting). **Jessica Metcalf:** Conceptualization (supporting); formal analysis (supporting); funding acquisition (equal); project administration (supporting); supervision (supporting); validation (equal); writing – review and editing (supporting).

## CONFLICT OF INTEREST STATEMENT

The authors of this manuscript do not have any competing interests to report.

## Data Availability

Data can be found in the following Dryad Repository: https://doi.org/10.5061/dryad.fbg79cp23.

## APPENDIX 1

We examined the relationship between rust prevalence and date of data collection to ensure that our methodology mitigated the confounding factor of temporality. A linear regression is depicted in red. No significant relationship was found (*p* = .415).

## APPENDIX 2

Having already established that flax is more likely to be found in a central range of 2900–3100 m, we next sought to further understand whether this effect was mediated by a flax population density dependence on elevation. We performed a linear regression and found no significant correlation between flax population density and elevation (estimate = .010043, *p* = .159).

## APPENDIX 3

Our observed slope aspect values (left: western slope aspect, right: southern slope aspect) were relatively evenly distributed across our range of sampled elevations.

## APPENDIX 4

A linear regression was performed in R, and overlaid over the data to determine the distribution of population connectivity across elevation. No significant relationship was observed.

## APPENDIX 5

Evidence demonstrating a lack of significant correlations between date of observation and any of the ecogeographical variables used in subsequent analyses.
